# Effects of dietary energy level on antioxidant capability, immune function and rectal microbiota in late gestation donkeys

**DOI:** 10.3389/fmicb.2024.1308171

**Published:** 2024-02-13

**Authors:** Yongmei Guo, Guolin Yin, Fang Hui, Xiaoyu Guo, Binlin Shi, Yanli Zhao, Sumei Yan

**Affiliations:** Key Laboratory of Animal Nutrition and Feed Science at Universities of Inner Mongolia Autonomous Region, College of Animal Science, Inner Mongolia Agricultural University, Hohhot, China

**Keywords:** gestation, dietary energy, donkey jennet, gut microbiota, oxidative stress, immunity

## Abstract

**Introduction:**

This study investigated the effects of dietary energy level on the antioxidant capability, immune function, and rectal microbiota in donkey jennets during the last 60 days of gestation.

**Methods:**

Fifteen pregnant DeZhou donkeys with age of 6.0 ± 0.1 years, body weight of 292 ± 33 kg, parity of 2.7 ± 0.1 parities and similar expected date of confinement (74 ± 4 days) were randomly allocated to three groups and feed three diets: high energy (10.92 MJ/kg, H), medium energy (10.49 MJ/kg, M), and low energy (9.94 MJ/kg, L).

**Results and Discussion:**

The serum activity of catalase (CAT), total superoxide dismutase (T-SOD), glutathione peroxidase (GSH-Px), and total antioxidant capacity (T-AOC) in group M was significantly higher, whereas the concentrations of malondialdehyde (MDA), interleukin 1 (IL-1), IL-2, and IL-6 were lower than those recorded for groups H and L (*p* ≤ 0.05). The dietary energy level significantly affected rectal microbial community structure in the jennet donkeys 35 days and 7 days before the parturition (*p* ≤ 0.05). The abundances of *norank_f_norank_o_Coriobacteriales* genus was significantly higher (*p* ≤ 0.05) in group H, and the abundances of *norank_f_norank_o_Mollicutes_RF39* and the *Candidatus_Saccharimonas* were higher in group L (*p* ≤ 0.05). The abundance of Fibrobacter in group M was significantly increased (*p* ≤ 0.05). The abundance of *norank_f_norank_o_Coriobacteriales* was positively correlated with average daily gain (ADG) and tumor necrosis factor-α (TNF-α) concentrations (*p* ≤ 0.05). The abundance of *norank_f_norank_o_Mollicutes_RF39* was positively correlated with IL-2 and IL-6 concentrations. The abundance of *Candidatus_Saccharimonas* was positively correlated with CAT, T-SOD and GSH-Px activities (*p* ≤ 0.05). The abundance of Fibrobacter was positively correlated with CAT and T-SOD activities (*p* ≤ 0.05), but negatively correlated with IL-2 concentration (*p* ≤ 0.05). In conclusion, an appropriate dietary with an energy content of 10.49 MJ/kg for jennet donkeys during late gestation increased the prenatal antioxidant capacity, reduced inflammatory cytokines, and promoted fetal growth, and these changes were related to diet-induced changes in rectal microbiota compositions.

## 1 Introduction

The market for donkey meat and milk continues growing in China as consumers pursue healthier foods (Liang et al., 2022). However, intensive donkey farming systems are not as well developed as those for other farm animal species. The feeding standards and corresponding supporting management systems have not been fully established. Gestation in donkey jennets, especially in the last quarter of pregnancy (3 months), is a critical period in achieving a successful reproduction outcome. The nutritional status and immune function of pregnant jennets at this stage of pregnancy is closely related to dietary energy level, and can determine foal birth weight. In dairy cows overfeeding during the late stage of pregnancy can result in oxidative stress and inflammation (Zhou et al., 2015), and similar results have been shown in ewes 7 days (d) before parturition (Moradi et al., 2021). However, the effects of dietary energy levels on antioxidant capability and immune function in donkeys during late pregnancy has not been reported.

Gut microbes are an “additional genome” of the host and associated with a host’s health through multiple pathways. Diet is a predominant factor that determines the gut microflora in animals. Changes in gut bacterial species are usually described using the Firmicutes/Bacteroidetes ratio as a marker of microbiome dynamics, and an increase in the Firmicutes/Bacteroidetes ratio is associated with high dietary energy levels in animal and human models (Malesza et al., 2021). Previous research found that a high-energy diet can increase lipopolysaccharides derived from the intestinal microbiota, and these derivatives act as inflammation trigger (Creely et al., 2007). The pro-inflammatory response is mediated by activating the nuclear factor, kappa-B pathway, that is involved in an over-expression of pro-inflammatory cytokines, such as tumor necrosis factor-α (TNF-α), interferon-γ, and inducible NO synthase (Dalvi et al., 2017). Due to the increased expression of the latter, the overproduction of nitric oxide (NO) is followed by the accumulation of reactive nitrogen species, and this is addition to reactive oxygen species (ROS) production (Tapias et al., 2017). Therefore, dietary energy level associated changes in the gut microbiota are closely related to the antioxidant and immune status of animals.

The donkey (*Equus africanus asinus*) has a well-developed hindgut structure. Therefore, microbial fermentation in the cecum and colon have always played an important role in equine species. Yin et al. (2021) study concluded that a dietary digestible energy (DE) level of 10.49 MJ/kg had the highest nutrient digestibility in female donkeys (Yin et al., 2021). It is hypothesized that higher or lower levels of dietary energy than 10.49 MJ/kg may result in changes in intestinal microflora and antioxidant status that are un-favor for donkey digestion systems.

Generally, the preferred sampling site for hindgut fermentation in animals is directly from the cecum or colon, but this requires animal slaughter or surgical cecum fistulation. In contrast, fecal samples contain most of cecal microbiota, and reflect the bacterial profile in the cecum (Kim and Isaacson, 2015; Stanley et al., 2015). Grimm et al. (2017) reported that changes in microbial communities in the cecum, colon and rectum followed similar patterns in response to dietary changes, thus changes in rectal microbiota can be used as an indicator of bacterial changes, energy metabolism and health of the host. Therefore, fecal samples were collected in this study to determine the correlations between rectal microflora diversity and antioxidant and immune indicators in late pregnant jennets in response to dietary energy levels. Changes in antioxidant capability, immune function, and rectal microbiota were also be measured along with foal birth weights and body size. The results will help to develop hypotheses to model the influence of dietary energy levels on gut microbiota and the healthy status of pregnant donkeys.

## 2 Materials and methods

The experiment was conducted in the Research Station of Inner Mongolia Agricultural University (Hohhot, China). The animal experimental procedures were performed in accordance with the National Standard Guidelines for Ethical Review of Animal Welfare (GB/T 35892-2018).

### 2.1 Experimental design, diet, and feeding management

Fifteen pregnant DeZhou jennets with an age of 6 ± 0.1 years, body weight of 292 ± 34 kg, parities of 2.7 ± 0.1 and an expected 74 ± 4 days to foaling were selected and randomly allocated to three group of five animals each. Three groups were then randomly allocated to three diets, containing a DE at 10.92 MJ/kg (H), 10.49 MJ/kg (M), or 9.94 MJ/kg (L) for the rest of gestation period. The ingredients and nutrient compositions of diets are shown in Table 1. The experiment lasted for 74 days (referred to as day −74 in reference to the days before foaling): the initial 14 days was for acclimation on the M diet and also to the environmental conditions, and in the following 60 days the groups were feed their respective diets and the required animal parameters for the 60 day experiment period measured. The jennets were kept in individual pens and fed twice a day at 07:30 and 14:00, respectively. The concentrate to forage ratio of the ration was maintained constant at 30 to 70. The jennets were free access to the diets and water.

**Table 1:** The ingredients and nutrient compositions of diets (air-dry basis).

	H^(4)^	M^(4)^	L^(4)^
Ingredients, %			
Millet straw	40.00	51.50	65.90
Alfalfa hay	16.00	8.00	2.00
Corn silage	14.00	10.50	2.10
Corn	17.44	14.44	11.34
Soybean meal	6.50	7.00	6.80
Corn gluten meal	0.20	1.70	2.80
Corn germ meal	0.30	1.30	1.40
DDGS	0.40	0.40	2.40
Wheat bran	0.40	1.50	2.40
Extruded full-fat soybean	2.90	1.80	1.00
Premix^(1)^	0.50	0.50	0.50
NaCl	0.37	0.37	0.37
CaCO_3_	0.33	0.33	0.33
CaHPO_4_	0.66	0.66	0.66
Total	100.00	100.00	100.00
Nutrient composition (%)			
DE (MJ/kg)^(2)^	10.92	10.49	9.94
DM^(3)^	87.09	87.21	87.30
C*p*^(3)^	11.55	11.52	11.50
EE^(3)^	2.91	2.81	2.95
NDF^(3)^	45.32	47.34	49.59
ADF^(3)^	27.07	27.65	28.36
Ca^(3)^	0.96	0.96	1.01
P^(3)^	0.31	0.31	0.32

1. Per kilogram of premix provided the following: Vitamin A 1,200,000 IU; Vitamin D 250,000 IU; Vitamin E 3,000 IU; Fe 4 g; Cu 1.6 g; Zn 12 g; Mn 12 g; I 72 mg; Se 60 mg; Co 100 mg.2. Digestible energy content (DE) was estimated using acid-insoluble ash in a donkey digestive experiment.3. DM: Dry matter; CP: Crude protein; EE: Ether extract; NDF: Neutral detergent fibre; ADF: Acid detergent fibre; Ca: Calcium; P: Phosphorus.4. H, M, and L refer to high digestible energy (10.92 MJ/kg), medium energy (10.49 MJ/kg), and low energy (9.94 MJ/kg) of the diets.

### 2.2 Measurements and sample collection

Feed offered and refusal were recorded daily for the experimental period. The amount of feed offered was adjusted daily to allow for 5% of refusals on the next day, so that voluntary feed intake could be measured. The average dry matter feed intake (DMI) for the whole experimental period was calculated.

The jennets were weighed before the morning feeding on days −60 and − 7 to calculate the average daily gain (ADG) over the 54 day period. Foal birth weight and sizes were recorded immediately after birth. Body length was the distance from the anterior margin of the scapula to the end of the sciatic bone; body height was the vertical distance from the top of withers to the ground; heart girth was the distance around the rib cage directly behind the forelimb; tube girth is the circumference of the finest part of the left forelimb cannon bone.

Fecal samples were collected via the rectum from each animal before the morning feeding on days −35 and − 7 and sample placed into sterile cryopreservation tubes. All samples were immediately snap frozen in liquid nitrogen and stored at −80°C until analysed. Blood samples (each 10 mL) were obtained via jugular puncture before the morning feeding on days −35, −21, −14, −7, and − 1. Serum was collected by centrifuging the blood tubes at 2054 × g for 10 min and the serum stored at −20°C.

### 2.3 Chemical assays

The serum concentrations of glucose (GLU), triglycerides (TG), cholesterol (CHO) and β-hydroxybutyric acid (BHBA) was analyzed by an automatic biochemical analyzer (Hitachi Co., Ltd., Tokyo, Japan, L-8900) using commercial kits (Lepu Diagnostics Co., Ltd., Beijing, China). The concentrations of non-esterified fatty acids (NEFA) and malondialdehyde (MDA) and the activity of catalase (CAT), total antioxidant capacity (T-AOC), total superoxide dismutase (T-SOD), glutathione, and peroxidase (GSH-Px) was analyzed using commercial kits (Nanjing Jiancheng Bioengineering Institute, Nanjing, China). The concentrations of interleukin1 (IL-1), interleukin 2 (IL-2), interleukin 6 (IL-6), and TNF-α were assayed using enzyme-linked immunosorbent assay (ELISA) kits (Shangbao Biotechnology Co., Ltd., Shanghai, China). All analyses were carried out according to the manufacturer’s protocols.

### 2.4 16S rRNA gene sequencing and operational taxonomic units picking

Total microbial DNA in five fecal samples from each dietary treatment were extracted using the E.Z.N.A.^®^ soil DNA kit (Omega Bio-tek, Norcross, GA, United States) according to the manufacturer’s protocol. The final DNA concentration and OD values at 260 nm and 280 nm were determined using a NanoDrop 2000 UV–vis spectrophotometer (Thermo Scientific, Wilmington, NC, United States), and DNA quality was checked using 1% agarose gel electrophoresis. Negative extraction controls were included in duplicates to control for extraction contamination. The extracted DNA was stored at −20°C until sequenced.

All PCR amplification and sequencing steps were carried out by Majorbio Bio-Pharm Technology Co. Ltd. (Shanghai, China). Libraries of 16S rRNA gene sequence were generated using a two-step PCR protocol. The V3-V4 region of the 16S rRNA gene was amplified using the universal bacterial and archaeal primers 338F (5’-ACTCCTACGGGAGGCAGCAG-3′) and 806R (5’-GGACTACHVGGGTWTCTAAT-3′) in the first step of PCR. The PCR reaction mix was 20 μL, including 4 μL of 5× FastPfu buffer, 2 μL of 2.5 mM dNTPs, 0.8 μL of each primer (5 μM), 0.4 μL of FastPfu polymerase and 10 ng of template DNA. The PCR product was extracted from agarose gel and further purified using AxyPrep DNA Gel Extraction kit (Axygen Biosciences, Union City, CA, United States) and quantified using QuantiFluor^™^-ST (Promega, Madison, WI, United States) according to the protocol. Sequencing of 16S rRNA gene amplicons was carried out according to the manufacturer’s protocol using an Illumina MiSeq (2 × 300 bp) and the MiSeq Reagent kit v3 (Illumina).

Raw data was filtered and analyzed using QIIME (Quantitative Insights into Microbial Ecology, version 1.9.1) software, quality-filtered by Trimmomatic and merged by FLASH (Fast Length Adjustment of Short Reads). Low-quality reads were removed using the following criteria: (i) The reads were truncated at any site receiving an average quality score < 20 over a 50 bp sliding window, (ii) primers’ matching allowed 2-nucleotide mismatching, and reads containing ambiguous bases were removed, and (iii) sequences with an overlap longer than 10 bp were merged according to their overlap sequence. The assembled sequences were assigned to operational taxonomic units (OTUs) at 97% similarity cutoff using UPARSE (Highly Accurate OTU Sequences from Microbial Amplicon Reads, version 7.1, http://drive5.com/uparse/, 30 September 2013) and chimeric sequences were identified and removed using UCHIME (Chimera Prediction for Amplicon Sequencing). The OTUs were used for α-diversity indexes (Coverage, Chao, Shannon and Simpson) analysis, and OTUs were taxonomically analyzed by the Ribosomal Database Project Classifier algorithm (http://rdp.cme.msu.edu/, 30 September 2016). The rarefaction curves were analysis with Mothur v.1.21.1 to identify sequence depth. Principal coordinate analysis (PCoA) was measured using the Bray-Curtis distance with the R software suite.

### 2.5 Statistical analysis

The data of DMI, ADG, birth weight and body sizes were analyzed using the analysis of variance (ANOVA) in SAS (SAS Software, Version 9.1; SAS Institute, Cary, NC, United States) and Duncan’s multiple range test set at the 0.05 level of significance (*p* ≤ 0.05). Blood biochemical indicators, antioxidant and immune indicators were analyzed in a complete randomized design model for repeated measures using the Proc Mixed procedure in SAS. The Mixed model included fixed effects for treatment (DE), prenatal time (PT, days before foaling) and DE × PT interactions and the random effects of pen. The differences in bacterial diversity indexes (Coverage, Chao, Shannon and Simpson) at phylum and genus classification levels were analyzed using ANOVA and Duncan’s multiple range tests in SAS. Spearman correlations was used to correlate nutrient digestibility, blood antioxidant indexes, and blood immune indexes with the bacteria enrichment at the genus classification level using the pheatmap package in the R software suite. Data for diet nutrient digestibility was obtained from a previous study (Yin, et al., 2021, see attached Supplementary Table S1). The threshold |R| ≥ 0.5 and *p* ≤ 0.05 were considered as a significant for the Spearman correlations. Data was presented as mean and standard error of means (SEM).

## 3 Results

### 3.1 ADG, DMI, birth weight and body size

The initial body weight of the donkey jennets was not different among three groups. DMI (*p* = 0.042) and ADG (*p* = 0.014) in group H were significantly higher than those in groups M and L, with no significant differences between groups M and L (Table 2). The differences in the nutrient intakes were consistent with DMI.

**Table 2:** Effects of dietary energy level on DMI, ADG and nutrient intake of donkey jennets during late gestation.

	H	M	L	SEM	*p*-value
Initial body weight, kg /kg	296	292	292	12.35	0.952
ADG, kg	0.46^A^	0.38^B^	0.29^B^	0.037	0.014
DMI, kg/d	6.39^A^	6.03^B^	6.03^B^	0.099	0.042
FCR	12.65^B^	14.76^B^	18.82^A^	1.101	0.011
DE, MJ/d	69.83^A^	65.80^B^	65.81^B^	1.084	0.042
Intake, kg/d					
CP	0.74^A^	0.70^B^	0.70^B^	0.011	0.042
EE	0.19^A^	0.18^B^	0.18^B^	0.003	0.043
NDF	2.90^A^	2.73^B^	2.73^B^	0.045	0.042
ADF	1.73^A^	1.63^B^	1.63^B^	0.027	0.042

A, B: means with different superscripts within a row differ significantly (*p* ≤ 0.05).

All foals survived the prenatal feeding regimes. The birth weight (*p* = 0.020), body length (*p* = 0.008), and body height for M and L group foals were significantly greater in H group (*p* = 0.004) (Table 3).

**Table 3:** Effects of dietary energy level for donkey jennets during late gestation on the birth weight and body sizes of donkey foals.

	H	M	L	SEM	*p-*value
Birth weight, kg	27.1^B^	31.7^A^	31.5^A^	1.03	0.020
Body length, cm	54.5^B^	59.0^A^	61.4^A^	1.06	0.008
Body height, cm	83.3^B^	87.6^A^	86.2^A^	0.82	0.004
Heart girth, cm	68.8	72.5	68.1	1.32	0.067
Tube girth, cm	10.8	10.9	11.2	0.17	0.385

Means with different superscripts within a row differ significantly (*p* ≤ 0.05). H, M, and L refer to high digestible energy (10.92 MJ/kg), medium energy (10.49 MJ/kg), and low energy (9.94 MJ/kg) of the diets.

### 3.2 Serum biochemical indicators

GLU (*p* < 0.001) and CHO (*p* < 0.001) concentrations for groups H and M were significantly higher than group L, the CHO concentration for group H was significantly higher than that of group M (*p* < 0.001) (Table 4). BHBA (*p* < 0.001) and NEFA (*p* < 0.001) concentrations were significantly lower in group M than in groups H and L, and group H was significantly lower than group L. The concentration of TG was significantly higher in group H than in groups M and L (*p* < 0.01). Prior to parturition, there was a significant increase in TG, GLU, BHBA, and NEFA concentrations (*p* < 0.05).

**Table 4:** Effects of dietary energy level on the biochemical indexes in serum of donkey jennets during late gestation.

DE	PT	GLU (mmol/L)	BHBA (mmol/L)	NEFA (umol/L)	CHO (mmol/L)	TG (mmol/L)
H	d-35	3.44^cdef^	0.20^de^	125^h^	3.60	1.08^cd^
	d-21	3.40^def^	0.22^bc^	274^gf^	3.61	1.17^bc^
	d-14	3.65^bcd^	0.19^e^	1004^bc^	3.60	1.19^bc^
	d-7	3.63^bcd^	0.20^de^	939^cd^	3.58	1.33^b^
	d-1	3.97^a^	0.22^bc^	1454^a^	3.83	1.66^a^
M	d-35	3.32^ef^	0.19^e^	102^h^	3.42	0.87^d^
	d-21	3.68^bc^	0.20^de^	219^g^	3.52	0.97^cd^
	d-14	3.41^def^	0.21^cd^	517^e^	3.09	0.95^cd^
	d-7	4.03^a^	0.20^de^	915^d^	3.03	1.00^cd^
	d-1	3.86^ab^	0.22^bc^	956^cd^	3.15	1.16^bc^
L	d-35	3.00^g^	0.21^cd^	123^h^	2.84	0.98^cd^
	d-21	3.28^f^	0.21^cd^	332^f^	3.02	1.00^cd^
	d-14	3.56^cde^	0.23^b^	1059^b^	2.96	0.96^cd^
	d-7	3.24^f^	0.23^b^	1038^b^	3.10	1.11^bcd^
	d-1	3.86^ab^	0.25^a^	1478^a^	2.79	1.12^bc^
SEM		0.074	0.004	23.45	0.112	0.075
Main effects						
DE	H	3.62^A^	0.21^B^	762^B^	3.65^A^	1.28^A^
	M	3.69^A^	0.20^C^	557^C^	3.24^B^	0.98^B^
	L	3.42^B^	0.23^A^	809^A^	2.91^C^	0.95^B^
PT	d-35	3.25^D^	0.20^C^	119^E^	3.29	1.05^B^
	d-21	3.45^C^	0.21^B^	282^D^	3.38	1.07^B^
	d-14	3.55^C^	0.21^B^	859^C^	3.22	1.02^B^
	d-7	3.74^B^	0.21^B^	1112^B^	3.24	0.96^B^
	d-1	3.90^A^	0.23^A^	1174^A^	3.26	1.29^A^
*p*-value	PT	<0.001	<0.001	<0.001	0.395	<0.001
	DE	<0.001	<0.001	<0.001	<0.001	<0.001
	PT*DE	<0.001	<0.001	<0.001	0.190	<0.001

A–D means with different superscripts within a column differ significantly (*p* ≤ 0.05). DE: Dietary digestible energy levels; PT: prenatal time (days before foaling) (*p* ≤ 0.05). a–g Means with different superscripts within a column indicate significant differences in the interaction between DE and PT (*p* ≤ 0.05). H, M, and L refer to high digestible energy (10.92 MJ/kg), medium energy (10.49 MJ/kg), and low energy (9.94 MJ/kg) of the diets.

There were significant interactions between DE and PT for GLU, BHBA, NEFA, and TG concentrations (*p* < 0.001). GLU was significantly higher on day −1 in all three groups, on day −7 in group M, but lower on day −35 in group L, compared with the other combinations of DE and PT (*p* < 0.001). BHBA concentrations was greatest on day −1 in group L, but lower on day −14 in group H and d − 35 in group M (*p* < 0.001). NEFA concentrations were greater on d − 1 in groups H and L, but lower on day −35 in all three groups (*p* < 0.001). The highest concentration of TG was observed on day −1 in group H, followed by day −7 in group H, on day −1 in groups M and L, with significantly lower concentrations on day −35 in group M (*p* < 0.001).

### 3.3 Serum blood antioxidants

The serum concentrations of the measured antioxidant indicators are shown in Table 5. CAT, T-SOD, GSH-Px, and T-AOC concentrations in group M were significantly higher than those in groups H and L (*p* < 0.001), while MDA concentration showed the opposite pattern. Approaching parturition, CAT, T-SOD, GSH-Px, and T-AOC concentrations decreased significantly, but MDA concentrations showed an increase (*p* < 0.001).

**Table 5:** Effects of dietary energy level on the antioxidant indexes in serum of donkey jennets during late gestation.

DE	PT	CAT (U/mL)	T-AOC (U/mL)	T-SOD (U/mL)	GSH-Px (U/mL)	MDA (nmol/mL)
H	d-35	2.97^cd^	2.22^b^	66.71^ab^	388^abc^	2.72
	d-21	2.77^de^	1.45^cde^	52.97^e^	377^bcd^	2.55
	d-14	2.43^defg^	1.22^def^	51.80^e^	370^cd^	3.65
	d-7	1.77^hi^	0.72^f^	54.97^cde^	334^cde^	4.93
	d-1	1.25^ji^	0.91^ef^	55.52^cde^	280^ef^	5.22
M	d-35	4.22^a^	2.77^a^	70.45^a^	665^a^	2.63
	d-21	3.84^ab^	1.79^bc^	63.77^abc^	455^ab^	2.65
	d-14	2.52^def^	1.17^def^	63.46^abc^	390^abc^	2.65
	d-7	1.86^gh^	1.11^def^	65.67^ab^	373^bcd^	3.38
	d-1	2.00^fgh^	1.17^def^	53.80^de^	333^cde^	3.91
L	d-35	3.42^bc^	1.67^cd^	66.04^ab^	456^ab^	3.35
	d-21	2.79^de^	1.25^def^	62.20^abcd^	327^cdef^	3.53
	d-14	2.21^efgh^	1.05^ef^	60.44^bcde^	322^cdef^	3.47
	d-7	2.33^efgh^	1.05^ef^	60.67^bcde^	294^def^	5.43
	d-1	0.93j	0.86^f^	38.67^f^	247^f^	4.54
SEM		0.193	0.175	2.824	26.48	0.383
Main effects						
DE	H	2.24^B^	1.30^B^	56.39^B^	350^B^	3.81^A^
	M	2.89^A^	1.62^A^	63.43^A^	403^A^	3.00^B^
	L	2.35^B^	1.17^B^	57.61^B^	330^B^	4.10^A^
PT	d-35	3.54^A^	1.95^A^	67.74^A^	433^A^	2.83^B^
	d-21	2.98^B^	1.63^B^	59.65^B^	349^B^	2.91^B^
	d-14	2.39^C^	1.04^C^	58.57^B^	361^B^	3.26^B^
	d-7	2.14^C^	0.96^C^	60.44^B^	359^B^	4.23^A^
	d-1	1.41^D^	1.21^C^	49.33^C^	303^C^	4.56^A^
*p*-value	PT	<0.001	<0.001	<0.001	<0.001	<0.001
	DE	<0.001	<0.001	<0.001	<0.001	<0.001
	PT*DE	<0.001	<0.001	<0.001	0.003	0.182

A–D Means with different superscripts within a column differ significantly (*p* ≤ 0.05). DE: Dietary digestible energy levels; PT: prenatal time (days before foaling) (*p* ≤ 0.05). a–g Means with different superscripts within a column indicate significant differences in the interaction between DE and PT (*p* ≤ 0.05). H, M, and L refer to high digestible energy (10.92 MJ/kg), medium energy (10.49 MJ/kg), and low energy (9.94 MJ/kg) of the diets.

There were significant interactions between DE and PT and CAT, T-SOD, GSH-Px, and T-AOC serum levels (*p* < 0.001). CAT activity was greater on days −35 and − 21 in group M, but lower on day −1 in groups H and L, and day −7 in group H (*p* < 0.001). T-AOC concentration was greater on day −35 in group M, but lower on days −14, −7, and − 1 in all three groups (*p* < 0.001). T-SOD concentration was greater on day −35 in all three groups, and on day −7 in group M, but lower on day −1 in group L (*p* < 0.001). GSH-Px serum level was greater on days −35, −21, and − 14 in group M, but lower on day −1 in group H and on days −21, −14, −7, and − 1 in group L (*p* = 0.003).

### 3.4 Serum immune indicators

The concentration of IL-1 (*p* < 0.001) and IL-2 (*p* < 0.001) in group M were significantly lower than those in group L, and group L were significantly lower than group H (Table 6). The concentration of IL-6 (*p* < 0.001) and TNF-α (*p* < 0.001) in group M were significantly lower than in groups H and L, and there was no difference between groups H and *L. prior* to parturition, the concentration of TNF-α increased significantly (*p* = 0.001).

**Table 6:** Effects of dietary energy level on the immune indexes in serum of donkey jennets during late gestation.

DE	PT	IL-1 (pg/mL)	IL-2 (pg/mL)	IL-6 (pg/mL)	TNF-α (pg/mL)
H	d-35	13.49^bc^	11.47^bc^	126	38.01^cde^
	d-21	13.81^b^	9.26^def^	127	50.49^a^
	d-14	17.84^a^	9.87^cd^	141	38.18^cde^
	d-7	14.02^b^	12.08^ab^	122	53.50^a^
	d-1	12.04^bcd^	13.41^a^	119	38.82^bcd^
M	d-35	9.62^e^	6.76^g^	94	27.75^e^
	d-21	9.62^e^	9.47^cd^	77	32.74^de^
	d-14	9.99^de^	7.67^fg^	99	33.96^cd^
	d-7	10.92^de^	8.31^defg^	93	34.10^de^
	d-1	11.04^de^	8.04^efg^	92	33.26^de^
L	d-35	11.47^cde^	9.34^def^	98	35.68^de^
	d-21	11.12^de^	9.97^cd^	139	29.85^de^
	d-14	10.68^de^	11.40^bc^	129	40.19^bcd^
	d-7	11.48^cde^	9.49^de^	112	48.86^ab^
	d-1	11.81^bcde^	9.76^cde^	114	46.42^abc^
SEM		0.714	0.553	9.79	3.321
Main effects					
DE	H	14.24^A^	10.82^A^	127^A^	44.23^A^
	M	10.24^C^	8.05^C^	91^B^	32.36^B^
	L	11.31^B^	9.99^B^	117^A^	40.23^A^
PT	d-35	11.53	9.19	106	33.81^C^
	d-21	11.51	9.57	114	37.69^BC^
	d-14	12.84	9.65	120	37.44^BC^
	d-7	12.14	9.96	109	40.59^AB^
	d-1	11.63	10.40	108	45.11^A^
*p*-value	PT	0.117	0.095	0.395	0.001
	DE	<0.001	<0.001	<0.001	<0.001
	PT*DE	<0.001	<0.001	0.201	<0.001

A–D Means with different superscripts within a column differ significantly (*p* ≤ 0.05). DE: Dietary digestible energy levels; PT: prenatal time (days before foaling) (*p* ≤ 0.05). a–g Means with different superscripts within a column indicate significant differences in the interaction between DE and PT (*p* ≤ 0.05). H, M, and L refer to high digestible energy (10.92 MJ/kg), medium energy (10.49 MJ/kg), and low energy (9.94 MJ/kg) of the diets.

There were significant interactions between DE and PT and IL-1, IL-2, and TNF-α concentrations (*p* < 0.001). The IL-1 concentration was greater on day −14 in group H than on the other days (*p* < 0.001). The concentration of IL-2 was greater on days −7 and − 1 in group H, and lower on days −35, −14, −7, and − 1 in group M (*p* < 0.001). Elevated TNF-α concentrations were observed on days −21 and − 7 in group H, and lower concentrations were measured on days −35, −21, −7 and − 1 in group M, on days −35 and − 7 in group H, and on days −35 and -21in group L (*p* < 0.001).

### 3.5 Rectal microbiota diversity

#### 3.5.1 Sampling depth

**Figure 1:**
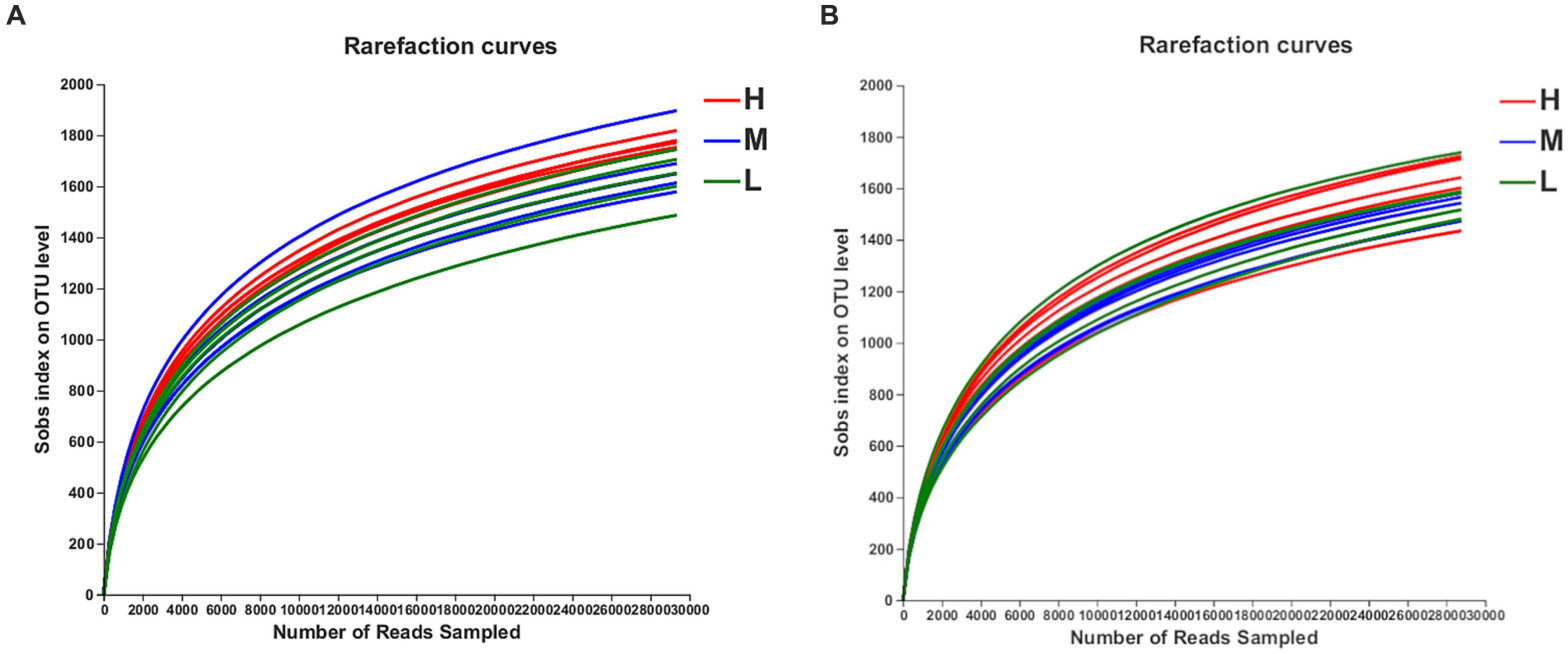
The OTU rarefaction curves of rectal microbiota in pregnant donkey jennets at 35 day (d-35, A) and 7 day (−7, B) before foaling. Curves were drawn using the least sequenced sample as upper limit for the rarefactions. Red curves and H: high dietary energy (10.92 MJ/kg); Blue curves and M: medium dietary energy (10.49 MJ/kg); Green curves and L: low dietary energy (9.94 MJ/kg).

On days −35, 2,673, 2,588, and 2,590 OTUs were separately obtained, on the basis of 97% species similarity, from rectal samples in groups H, M, and L, respectively (Table 7). The average of 61,758, 61,528, and 54,499 sequences were recorded for each sample from the groups H, M and L, respectively. On day −7, 2,530, 2,381, and 2,485 OTUs were obtained, and an average of 56,273, 53,261, and 57,794 sequences were measured for each sample from groups H, M, and L, respectively. Both the rarefaction curves (Figure 1) and the high coverage values (Table 8) obtained showed that sampling depths met the requirements for estimating bacterial diversity.

**Table 7:** Sequence data of rectal microbiota.

	H	M	L
**d-35**			
OTUs	2,673	2,588	2,590
Optimized sequences	170,192	162,570	144,609
Average optimized sequences of sample	61,758	61,528	54,499
Average length of optimized sequence	414	415	415
**d-7**			
OTUs	2,530	2,381	2,485
Optimized sequences	182,597	141,669	177,524
Average optimized sequences of sample	56,273	53,261	57,794
Average length of optimized sequence	412	414	413

**Table 8:** The α-diversity indexes for rectal microbiota in donkey jennets during late gestation.

	H	M	L	SEM	*p*-value
**d-35**					
Shannon	6.29^A^	6.16^AB^	5.89^B^	0.091	0.039
Chao	2253^A^	2143^AB^	2061^B^	37.305	0.017
Simpson	0.004^B^	0.005^B^	0.008^A^	0.001	0.017
Coverage	0.99	0.99	0.99	0.0004	0.224
**d-7**					
Shannon	6.12^A^	5.87^B^	5.81^B^	0.062	0.009
Chao	2120^A^	2007^B^	2033^B^	27.199	0.031
Simpson	0.006^B^	0.009^AB^	0.013^A^	0.001	0.025
Coverage	0.99	0.99	0.99	0.0005	0.472

A–D means with different superscripts within a row differ significantly (*p* ≤ 0.05). d-35, d-7: days before foaling. H, M, and L refer to high digestible energy (10.92 MJ/kg), medium energy (10.49 MJ/kg), and low energy (9.94 MJ/kg) of the diets.

#### 3.5.2 Rectal microbiota diversity indexes

On day −35, Shannon and Chao indexes were significantly greater in group H than those recorded in group L (*p* ≤ 0.05), and there was no significant differences in Shannon and Chao indexes between groups H and M, as well as, between groups M and L (Table 8). Simpson index was significantly higher in group L than those in groups H and M (*p* = 0.017), and there was no significant difference between groups H and M. On day −7, Shannon and Chao indexes were significantly greater in group H than those observed in groups M and L (*p* ≤ 0.05), and there was no significant difference between groups M and L. Simpson index was significantly greater in group L than that in group H (*p* = 0.025), and there was no significant difference between groups M and H, and between groups M and L.

**Figure 2:**
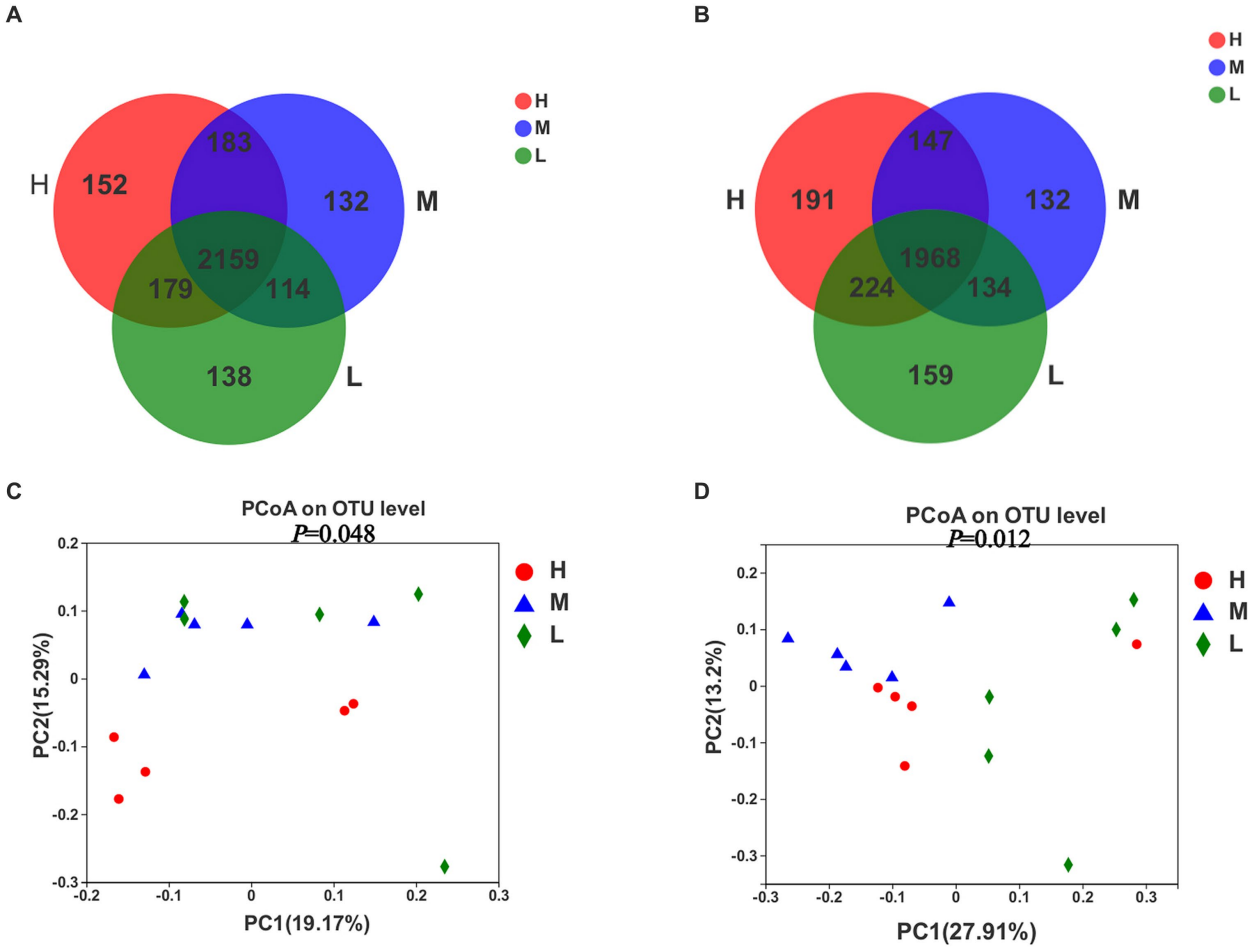
Venn plot of OTUs showing the percent of observations for each OTU (> 0.5%) **(A,B)** and Principal coordinate analysis (PCoA) of bacterial OTUs **(C,D)** in the rectal samples of pregnant donkey jennets at 35 days (d-35, **A,C**) and 7 days (−7, **B,D**) before foaling. Red color and H: high dietary energy (10.92 MJ/kg); Blue color and M: medium dietary energy (10.49 MJ/kg); Green color and L: low dietary energy (9.94 MJ/kg).

A total of 3,057 OTUs were obtained for all the samples on day −35 (Figure 2A), of which 2,159 defined as the core OTUs present in all three groups. The core OTUs consisted of 70.6% of the total OTUs. In addition, 152, 132, and 138 OTUs were uniquely identified in groups H, M and L, respectively. On day −7 (Figure 2B), a total of 2,955 OTUs were obtained for all the samples, of which the core OTUs were 1,968, accounting for 66.6% of the total OTUs. In addition, 191, 132, and 159 OTUs were uniquely identified in groups H, M, and L, respectively. The PCoA plots demonstrated dissimilarities between groups H and M, as well as between groups H and L on day −35 (Figures 2C,D), but no specific clustering was identified between groups M and L. However, the results of rectal microbiota sampled on day −7 showed dissimilarities between groups H, M, and L. Samples in group M occupied the top left of PC1, samples in group H occupied the bottom left of PC1, and samples in group L occupied the right side of PC1.

#### 3.5.3 Taxonomic classifications levels of the bacterial communities

At the phylum level (Table 9), on day −35, Firmicutes (> 56% of the total), Bacteroidetes, Spirochaetes, Kiritimatiellaeota, Actinobacteria, Patescibacteria, Fibrobacteres, Proteobacteria, Verrucomicrobia, Tenericutes, were the predominant phyla (> 99% of total), and overall there were not differences between three treatment groups except for Verrucomicrobia. The abundance of Verrucomicrobia was significantly lower in group M than those in groups H and L (*p* = 0.013). The abundance of these predominant phyla (> 99% of the total) did not change significantly by day −7. The abundance of Patescibacteria and Tenericutes in group M were significantly lower than those of groups H and L, whereas the abundance of Fibrobacteres was higher (*p* < 0.05). The abundance of Actinobacteria in group L was significantly lower than in group H (*p* = 0.049), but no significant differences was found between groups M and H, and between M and L.

**Table 9:** Effects of dietary energy level on the relative abundance (%) of rectal microbiota at Phylum level in donkey jennets during late gestation.

Phylum	H	M	L	SEM	*p*-value
**d-35**					
Firmicutes	56.39	56.68	60.24	3.265	0.659
Bacteroidetes	29.81	31.05	27.50	2.258	0.387
Spirochaetes	4.22	5.00	3.39	0.831	0.419
Kiritimatiellaeota	3.79	2.67	3.40	0.638	0.476
Actinobacteria	1.01	0.96	1.35	0.168	0.250
Patescibacteria	1.35	0.64	0.95	0.218	0.113
Fibrobacteres	0.84	1.02	0.75	0.270	0.777
Proteobacteria	0.81	0.79	0.91	0.174	0.876
Verrucomicrobia	0.86^A^	0.32^B^	1.08^A^	0.493	0.013
Tenericutes	0.41	0.38	0.40	0.181	0.761
**d-7**					
Firmicutes	69.74	63.16	61.80	3.089	0.193
Bacteroidetes	20.63	25.83	26.62	2.302	0.131
Spirochaetes	2.50	4.28	3.56	2.251	0.464
Kiritimatiellaeota	2.54	2.19	1.83	0.604	0.712
Patescibacteria	1.14^A^	0.53^B^	1.75^A^	0.413	0.008
Actinobacteria	1.31^A^	1.16^AB^	0.79^B^	0.136	0.049
Verrucomicrobia	0.62	0.95	1.03	0.715	0.691
Proteobacteria	0.59	0.91	0.56	0.547	0.990
Tenericutes	0.44^A^	0.24^B^	0.56^A^	0.160	0.039
Fibrobacteres	0.15^B^	0.41^A^	0.14^B^	0.157	0.037

A–D means with different superscripts within a row differ significantly (*p* ≤ 0.05). d-35, d-7: days before foaling. H, M, and L refer to high digestible energy (10.92 MJ/kg), medium energy (10.49 MJ/kg), and low energy (9.94 MJ/kg) of the diets.

**Figure 3:**
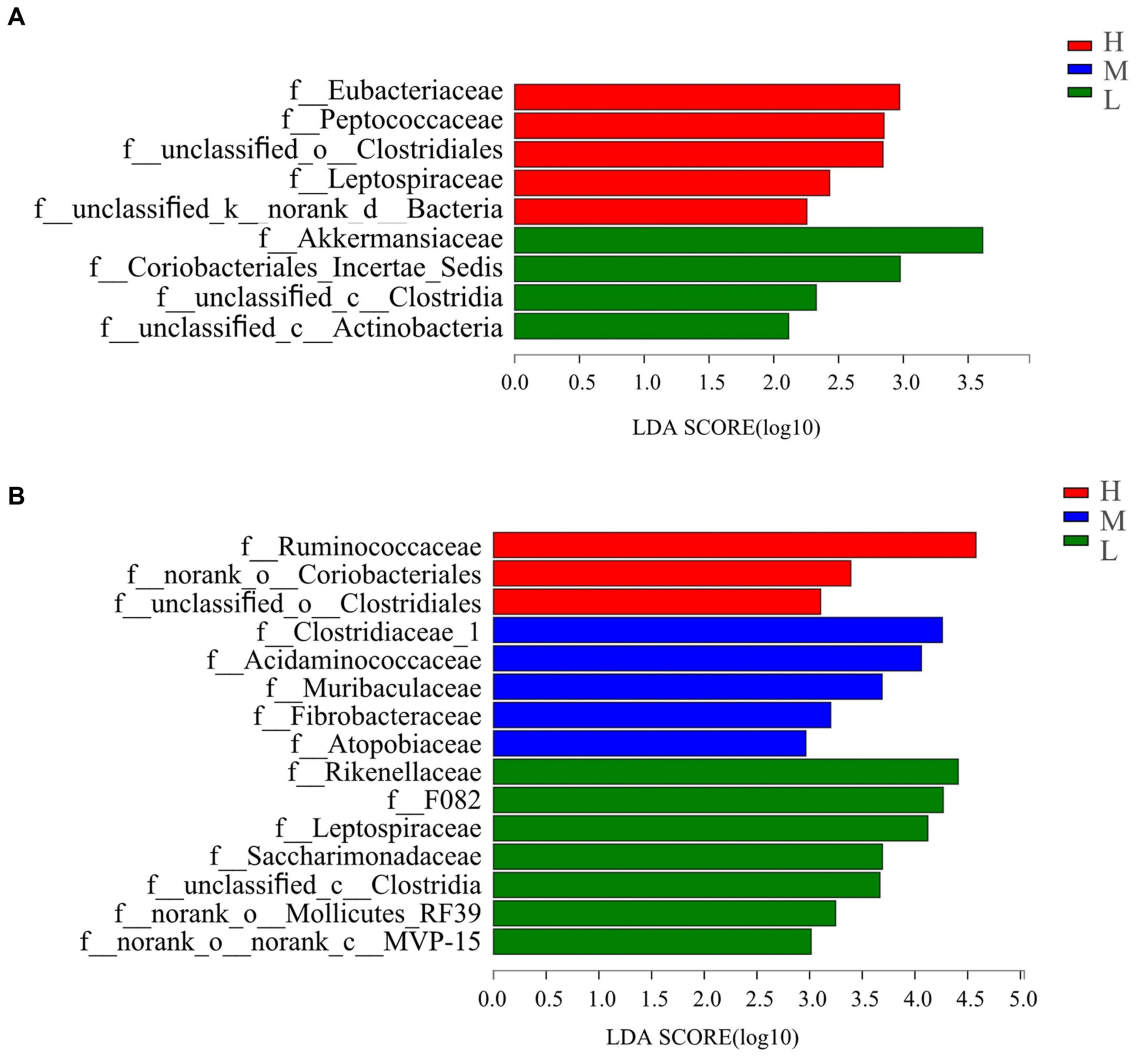
Bacterial families’ differentiation indicated by linear discriminant analysis (LDA) effect size (LEfSe) in rectal samples of pregnant donkey jennets at 5 days **(A)** and 7 days **(B)** before foaling. Red color and H: high dietary energy (10.92 MJ/kg); Blue color and M: medium dietary energy (10.49 MJ/kg); Green color and L: low dietary energy (9.94 MJ/kg).

Linear discriminant analysis (LDA, Figure 3A) used for bacterial family discrimination found the abundances of Eubacteriaceae, Peptococcaceae, unclassified_o_Clostridiales, Leptospiraceae and unclassified_k_norank_d_Bacteria were all significantly elevated on day −35 in group H compared with the abundances in groups M and L. The abundance Akermansiacea, Coriobacteriales_Incertae_Sedis, unclassified_c_Clostridia, and unclassified_c_Actinobacteria families were significantly higher on day −35 in group L compared the abundances in groups H and M.

On day −7 (Figure 3B), the abundances of Ruminococcaceae, norank_o_Coriobacteriales, and unclassified_o_Clostridiales were higher in group H than the abundances in groups M and L. However, the abundance of Clostridiaceae_1, Acidaminococcaceae, Muribaculaceae, Fibrobacteraceae, and Atopobiaceae were significantly higher in group M in comparison with those recorded in groups H and L. The abundances of Rikenellaceae F082, Leptospiraceae, Saccharimonadaceae, unclassified_c_Clostridia, norank_o_Mollicutes_RF39, and norank_o_norank_c_MVP-15, were significantly higher in group L than those for groups H and M.

**Figure 4:**
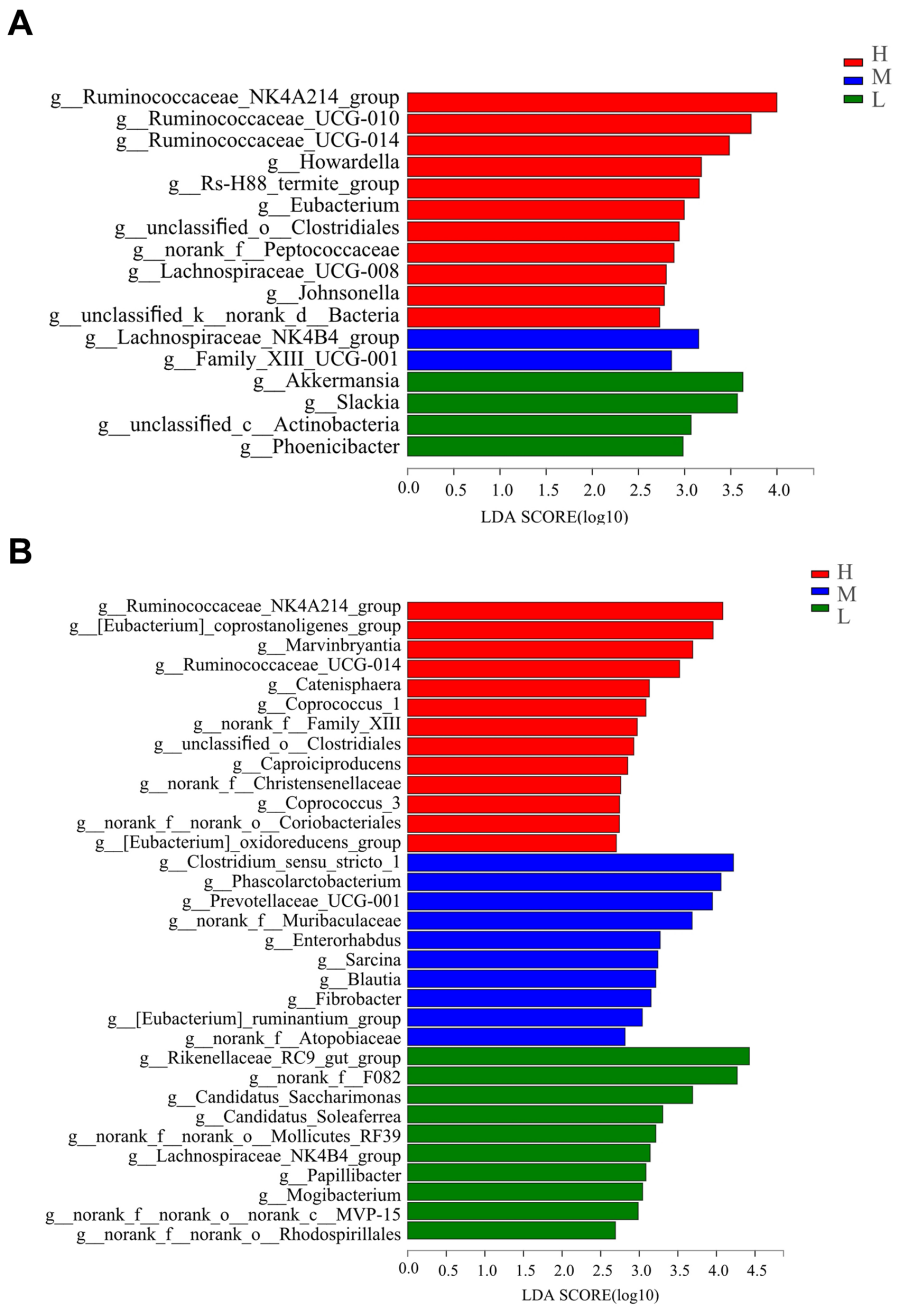
Bacterial genus differentiation indicated by linear discriminant analysis (LDA) effect size (LEfSe) in rectal samples of pregnant donkey jennets at 35 days **(A)** and 7 days **(B)** before foaling. Red color and H: high dietary energy (10.92 MJ/kg); Blue color and M: medium dietary energy (10.49 MJ/kg); Green color and L: low dietary energy (9.94 MJ/kg).

At the genus level (Figure 4A), the genera *Ruminococcaceae_NK4A214_group*, *Ruminococcaceae_UCG-010*, *Ruminococcaceae_UC-G-014*, *Howardella*, *Rs-H88_termite_group*, *Eubacterium*, *unclassified_o_Clostridiales*, *norank_f_Peptococcaceae*, *Lachnospiraceae_UCG-008*, *Johnsonella,* and *unclassified_k_norank_d_Bacteria* were greater on day −35 in group H compared with those in groups M and L. The abundances of *Lachnospiraceae_NK4B4_group* and *Family_XIII_UCG-001* were significantly higher in group M. The abundance of *Akkermansia*, *Slackia*, *unclassified_c_Actinobacteria,* and *Phoenicibacte* were significantly higher in group L than those recorded for the H and M treatments.

On day −7 (Figure 4B), the abundance of 13 genera in group H significantly increased compared with those for groups M and L, and the genera were *Ruminococcaceae_NK4A214_group*, *[Eubacterium]_coprostanoligenes_group, Marvinbryantia*, *Ruminococcaceae_UCG-014*, *Catenisphaera*, *Coprococcus_1*, *norank_f_Family_XIII*, *unclassified_o_Clostridiales*, *Caproiciproducens*, *norank_f_Christensenellaceae*, *Coprococcus_3*, *norank_f_norank_o_Coriobacteriales,* and *[Eubacterium]_oxidoreducens_group*. Groups H and L had significantly lower abundances of *Clostridium_sensustricto_1*, *Phascolarctobacterium*, *Prevotellaceae_UCG-001*, *norank_f_Muribaculaceae*, *Enterorhabdus*, *Sarcina*, *Blautia*, *Fibrobacter*, *[Eubacterium]_ruminantium_group*, and norank_f_Atopobiaceae generas than group M. The abundances of *Rikenella-ceae_RC9_gut_group*, *norank_f_F082*, *Candidatus Saccharimona*s, *Candidatus_Soleaferrea*, *norank_f_norank_o_Mollicutes_RF39*, *Lachnospiraceae_NK4B4_group*, *Papillibacter*, Mogibacterium, norank_f_norank_o_norank_-c_MVP-15, and *norank_f_norank_o_Rhodospirillales* in group L were significantly evaluated compared with those abundances for groups H and M.

#### 3.5.4 Spearman correlation analysis

**Figure 5:**
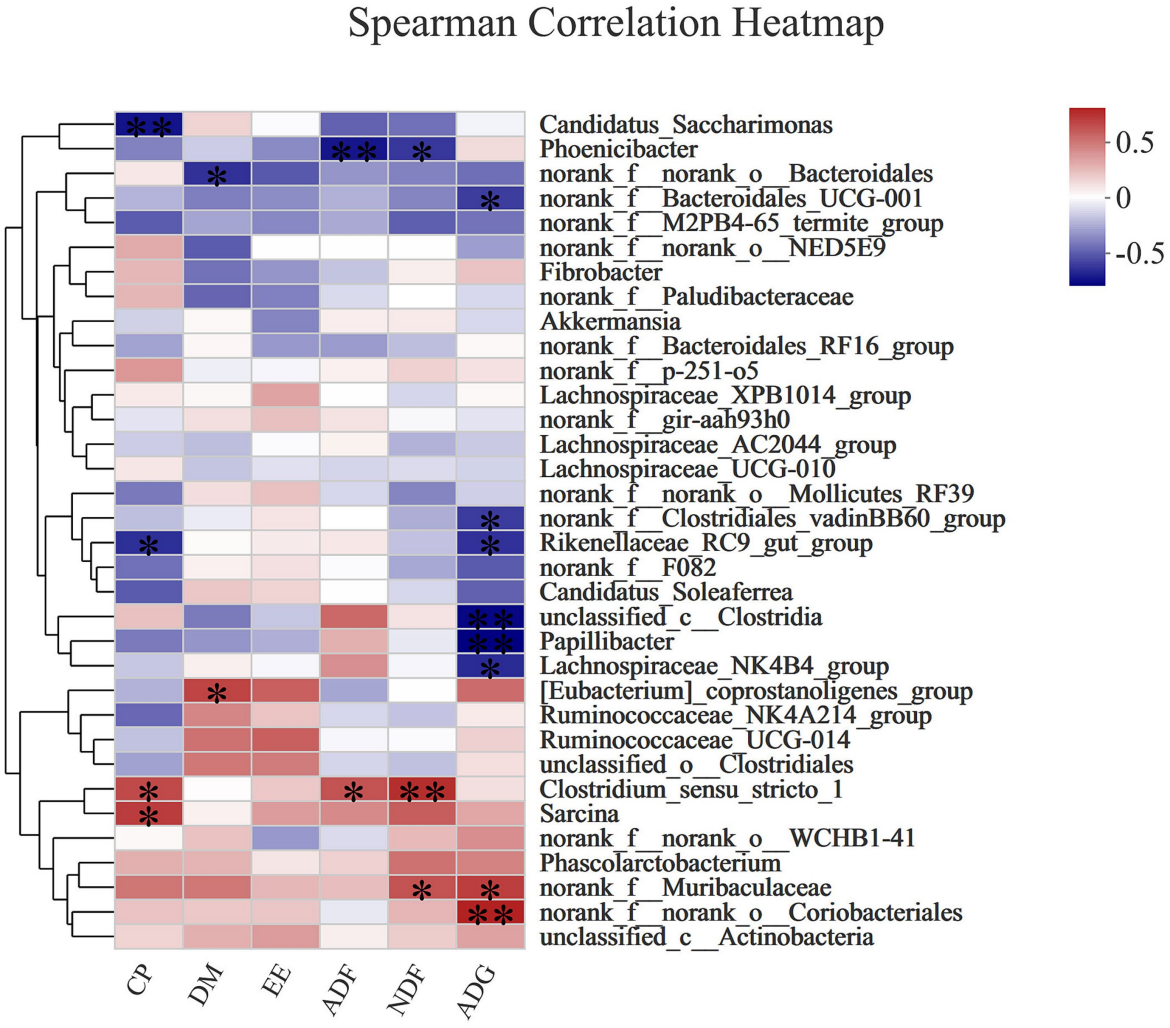
The Spearman correlation analysis of the rectal microbial composition at genus levels with nutrient (DM, EE, ADF, NDF) digestibility and ADG in donkey jennets during late gestation. Red color: positive correlation; Blue color: negative correlation. *: *p* ≤ 0.05; **: *p* ≤ 0.01.

Figure 5 shows that crude protein (CP) digestibility was positively correlated with *Clostridium_sensu_stricto_1* (Spearman R = 0.58027, Pcorr = 0.02334) and *Sarc*ina (Spearman R = 0.62366, Pcorr = 0.01298), but negatively correlated with *Rikenellaceae_RC9_gut_group* (Spearman R = −0.60395, Pcorr = 0.01711) and *Candidatus_Saccharimonas* (Spearman R = −0.6828, Pcorr = 0.00503). Dry matter (DM) digestibility was positively correlated with *[Eubacterium]_coprostanoligenes_group* (Spearman R = 0.59857, Pcorr = 0.0184), but negatively correlated with *norank_f_norank_o_Bacteroidales* (Spearman R = −0.5991, Pcorr = 0.01827). Acid detergent fiber (ADF) digestibility was positively correlated with *Clostridium_sensustricto_1* (Spearman R = 0.54857, Pcorr = 0.03422), but negatively correlated with *Phoenicibacte* (Spearman R = −-0.67389, Pcorr = 0.00587). Neutral detergent fiber (NDF) digestibility was positively correlated with *Clostridium sensustricto_1* (Spearman R = 0.66728, Pcorr = 0.00657) and *norank_f_Muribaculaceae* (Spearman R = 0.55167, Pcorr = 0.03301), but negatively correlated with *Phoenicibacte* (Spearman R = −-0.58537, Pcorr = 0.02187). The ADG was significantly correlated with *norank_f_Muribaculaceae* (Spearman R = 0.61649, Pcorr = 0.01438) and *norank_f_norank_o_Coriobacteriales* (Spearman R = 0.70927, Pcorr = 0.00307), but negatively correlated with *norank_f_Bacteroidales_UCG-001* (Spearman R = −0.56631, Pcorr = 0.02774), *norank_f_Clostridiales_vadinBB60_group* (Spearman R = −0.56808, Pcorr = 0.02715), *Rikenellaceae_RC9_gut_group* (Spearman R = −0.59857, Pcorr = 0.0184), *unclassified_c_Clostridia* (Spearman R = −0.71709, Pcorr = 0.00262), *Papillibacter* (Spearman R = −0.73836, Pcorr = 0.00167), and *Lachnospiraceae_NK-4B4_group* (Spearman R = −0.61721, Pcorr = 0.01423).

**Figure 6:**
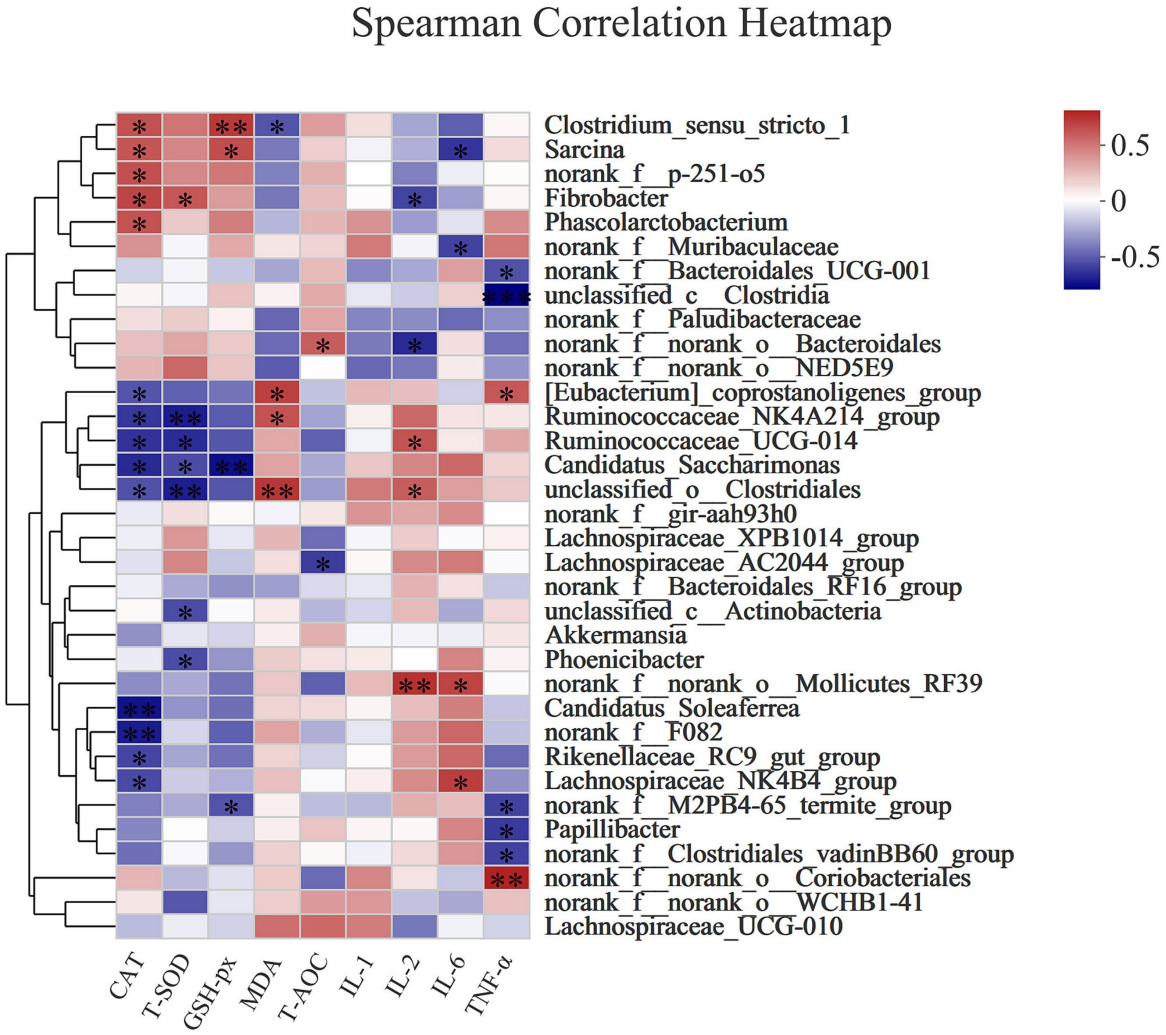
The Spearman correlation analysis of the rectal microbial composition at genus levels with antioxidant (CAT, T-SOD, GSH-Px, MDA, T-AOC) and immune (IL-1, IL-2, IL-6, and TNF-α) indexes in donkey jennets during late gestation. Red color: positive correlation; Blue color: negative correlation. *: *p* ≤ 0.05; **: *p* ≤ 0.01.

Figure 6 shows that CAT activity was positively correlated with *Clostridium_sensustricto_1* (Spearman R = 0.5716, Pcorr = 0.02601), *Sarcina* (Spearman R = 0.55571, Pcorr = 0.03149)*, norank_f_p-251-o5* (Spearman R = 0.57562, Pcorr = 0.02474), *Phascolarctobacterium* (Spearman R = 0.56838, Pcorr = 0.02706), and *Fibrobacter* (Spearman R = 0.61363, Pcorr = 0.01497), but negatively correlated with *[Eubacte-rium]_coprostanoligenes_group* (Spearman R = −0.51407, Pcorr = 0.04995), *Ruminococcaceae_NK4A214_group* (Spearman R = −0.60277, Pcorr = 0.01739), *unclassified_o_Clostridiales* (Spearman R = −0.5245, Pcorr = 0.04472), *Candidatus_Saccharimonas* (Spearman R = −0.63716, Pcorr = 0.01063), *Ruminococcaceae_UCG-014* (Spearman R = −0.61363, Pcorr = 0.01497), *Candidatus_Soleaferrea* (Spearman R = −0.70595, Pcorr = 0.00327), *norank_f_F082* (Spearman R = −0.67759, Pcorr = 0.00551), *Rikenellaceae_RC9_gut_group* (Spearman R = −0.55933, Pcorr = 0.03017), and *Lachnospiraceae_NK4-B4_grou*p (Spearman R = −0.54479, Pcorr = 0.03573). T-SOD was positively correlated with *Fibrobacter* (Spearman R = 0.55158, Pcorr = 0.03304), but negatively correlated with *Ruminococcaceae_NK4A214_group* (Spearman R = −0.67456, Pcorr = 0.00581), *Ruminococcaceae_UCG-014* (Spearman R = −0.63115, Pcorr = 0.01163), *Candidatus_Saccharimonas* (Spearman R = −0.53892, Pcorr = 0.03818), *unclassified_o_Clostridiales* (Spearman R = −0.66973, Pcorr = 0.00631), *unclassified_c_Actinobacteria* (Spearman R = −0.53748, Pcorr = 0.0388), and *Phoenicibacter* (Spearman R = −0.5382, Pcorr = 0.03849). GSH-Px activity was positively correlated with *Clostridium_sensustricto-1* (Spearman R = 0.64933, Pcorr = 0.0088) and *Sarcina* (Spearman R = 0.58423, Pcorr = 0.02219), but negatively correlated with *Candidatus_Saccharimonas* (Spearman R = −0.71685, Pcorr = 0.00263) and *norank_f_M2PB4-65_termite_group* (Spearman R = −0.5178, Pcorr = 0.04803). MDA concentration was positively correlated with *[Eubacterium]_coprostanoligenes_group* (Spearman R = 0.62705, Pcorr = 0.002621235), *Ruminococcaceae_NK4A214_group* (Spearman R = 0.56939, Pcorr = 0.02672), and *unclassified_o_Clostridiales* (Spearman R = 0.65917, Pcorr = 0.00752), but negatively correlated with *Clostridium_sensustricto_1* (Spearman R = −0.51399, Pcorr = 0.04999). T-AOC was positively correlated with *norank_f_norank_o_Bacteroidales* (Spearman R = 0.53134, Pcorr = 0.04152), but negatively correlated with *Lachnospiraceae_AC2044_group* (Spearman R = −0.58335, Pcorr = 0.02245).

IL-2 content was positively correlated with *Ruminococcaceae_UCG-014* (Spearman R = 0.56269, Pcorr = 0.02898), *unclassified_o_Clostridiales* (Spearman R = 0.52686, Pcorr = 0.0436), and *norank_f_norank_o_Mollicutes_RF39* (Spearman R = 0.68067, Pcorr = 0.00522), but negatively correlated with *norank_f_norank_o_Bacteroidales* (Spearman R = −0.63599, Pcorr = 0.01082) and *Fibrobacter* (Spearman R = −0.5645, Pcorr = 0.02836). IL-6 concentration was positively correlated with *Lachnospiraceae_NK4B4_group* (Spearman R = 0.63397, Pcorr = 0.01115) and *norank_f_norank_o_Mollicutes_RF39* (Spearman R = 0.61512, Pcorr = 0.01466), but negatively correlated with *Sarcina* (Spearman R = −0.60433, Pcorr = 0.01702) and dam*norank_f_Muribaculaceae* (Spearman R = −0.56836, Pcorr = 0.02706). TNF-α concentration was positively correlated with *[Eubacterium]_coprostanoligenes_group* (Spearman R = 0.55368, Pcorr = 0.03225) and *norank_f_norank_o_Coriobacteriales* (Spearman R = 0.73099, Pcorr = 0.00196), but negatively correlated with *norank_f_Bacteroidales_UCG-001* (Spearman R = −0.52302, Pcorr = 0.04544), *unclassified_c_Clostridia* (Spearman R = −0.7605, Pcorr = 0.001), *norank_f_M2PB4-65_termite_group* (Spearman R = −0.56829, Pcorr = 0.02709), *Papillibacter* (Spearman R = −0.58795, Pcorr = 0.02116), and *norank_f_Clostridiales_vadinBB60_group* (Spearman R = −0.56895, Pcorr = 0.02687).

## 4 Discussion

In late gestation, fetal growth rate is at its highest, demanding more nutrients from the dam, with glucose consumption by the fetus accounting for 46% of maternal glucose production (Weber et al., 2013). The growth and development of the fetus in the uterus occupies most of the space in the abdominal cavity, suppressing the gestating female’s feed intake (Du et al., 2018). Thus animals in late pregnancy are prone to chronic negative energy balance, leading to an increase in fat mobilization and release of large amounts of NEFA into the blood and liver (White, 2015). When NEFA is incompletely oxidized to ketones, forming BHBA, there is decreased production efficiency, but there is also increased blood ketone concentrations, as well as, as an increased risk of fatty liver disease (Weber et al., 2013). In the present study, the donkey jennets during the last two months of gestation showed an increase in serum concentrations of glucose, BHBA, and NEFA with the progression of gestation, and the concentrations reached the highest values prior to parturition. Our results conform to literature reports that donkey jennets during this late stage of pregnancy are subject to energy deficiency problems. However, the status of the negative energy balance in the donkey jennets can be modulated with increased dietary energy levels, with the jennets on the M diet having greater glucose levels but lower NEFA and BHBA concentrations compared to the donkeys on the H and L diets. These results indicate that feeding donkey jennets during the late stage of gestation with a diet containing an appropriate level of dietary energy, i.e., 10.49 MJ/kg, improved energy utilization in the body and reduced the impact of negative energy balance on serum parameters.

DMI and ADG generally increased with increasing dietary energy levels and in this experiment diet H had higher DMI and ADGs than diets M and L. However, birth weight and body sizes of the foals were the same on the M and L diets, and the foals from the jennets on the H diet had lower birth weights. The results indicate that the energy supplied from the M and L diets plus the body fat mobilization in the jennets could meet the energy demand for fetal growth, even at the lower level of dietary energy supplied in the present study. Studies in beef cattle have shown the importance of pre-partum dietary energy levels for neonatal calves on calf birth weight (Chen et al., 2022) with only severe maternal energy deficiency impairing the birth weight of the offspring. This was not the case in this donkey study.

β-oxidation of NEFA produces a large amounts of ROS, and excessive free radicals breaks the balance between oxidation and antioxidant systems, potentially leading to oxidative stress. Oxidative stress is an important cause of increased inflammatory responses and immune suppression (Sordillo and Aitken, 2009). This is likely to be one of the mechanisms for the increased occurrence of oxidative stress and immune suppression in undernourished animals in late stage of gestation. Therefore, it is crucial to increase dietary energy intake levels for dams in late gestation. However, the inevitable accumulation of excessive energy in the body induces cell enlargement and stress, with the release of inflammatory factors (Maury and Brichard, 2010; Meijer et al., 2011). Thus, an appropriate dietary energy intake during gestation is essential. Liang et al. (2020) reported that dairy cows with high body condition scores pre-partum were more likely to be susceptible to oxidative stress. In the present study, serum concentrations of MDA, IL-1, IL-2, and TNF-α were greater in group H, whereas the activities of CAT, T-AOC, and GSH-Px were lower, compared with the jennets on the M and L diets, indicating that the supply of a high level of dietary energy to the jennets during the late gestation can cause oxidative stress and inflammation. The negative energy balance in the jennets on the low energy diet also resulted in a detrimental effect on antioxidant capacity and immune responses. Therefore, the dietary energy level in group M was the more appropriate diet for donkey jennets during late gestation.

The present study found similar microbial diversities in donkey rectums on days 35 and 7 prior to parturition, as shown by the similar values for Shannon and Chao indexes. However, the microbial diversity changed with the dietary energy levels. The Shannon and Chao indexes in group H were higher, but Simpson index was lower than those in the other two groups, reflecting higher microbial richness and more diversified microbial community in pregnant jennets on the high-energy diet. Similarly, intestinal flora diversity was higher in obese horses compared to the flora diversity in lean horses (Biddle et al., 2018). Previous studies have shown that Firmicutes and Bacteroidetes are the dominant microbial communities in the guts of rodents, swine, horses, and cattle (Al Jassim and Andrews, 2009; Callaway et al., 2010; Holman and Chénier, 2014), and are similar to the results in this study. Firmicutes and Bacteroidetes are the main phyla, respectively, for fiber degradation and carbohydrate catabolism in the gastrointestinal tract of herbivores (Spence et al., 2006; Dearing and Kohl, 2017). Compared with group L, there was a greater abundance of Actinobacteria in group H as well as, a greater abundance of Actinobacteria in group M. Detailed analysis identified that *norank_o_Coriobacteriales* and *norank_f_norank_o_Coriobacteriales* were the main genera that resulted in the relative abundance of Actinobacteria in group H. The abundance of *norank_f_norank_o_Coriobacteriales* was positively correlated with ADG and serum TNF-α concentration in this study. Previous studies have shown that Actinobacteria were correlated with lipid content that promotes animal growth and development. These resultant bioactive substances participate in host metabolism, but their abundances increase the prevalence of inflammation (Bredholdt et al., 2007; Dougal et al., 2014). The mechanism of action of Actinobacteria includes the following three aspects: Firstly, acetic acid produced by Actinobacteria, when absorbed by the colon, crosses the blood–brain barrier to activate hypothalamic neurons, activating the parasympathetic nerves, stimulating the secretion of growth hormone that increases feed intake and body weight gain (Koeth et al., 2013; Warrier et al., 2015); Secondly, acetic acid stimulates adipocyte storage by stimulating islet B cells to release insulin (Vrieze et al., 2012); Thirdly, hydrogen sulfide produced by Actinobacteria increases intestinal wall permeability and impairs intestinal integrity, thus some harmful bacteria and their products in the intestine can pass into the intestinal mucosa, leading to increased levels of inflammation and decreased levels of anti-inflammatory factors, triggering a chronic inflammatory state and insulin resistance (Hui et al., 2012; Kilian, 2018). In addition, this experiment also reported the abundance of Firmicutes in group H was numerically increased compared to group M and L, and especially the genus of *[Eubacterium]_coprostanoligenes_group*. The abundance of *[Eubacterium]_coprostanoligenes_group* was positively correlated with DM digestibility and serum MDA and TNF-α concentrations, but negatively correlated with CAT activity. Therefore, the greater abundance of *[Eubacterium]_coprostanoligenes_group* may be one of the factors involved in lowering the concentrations of antioxidant indicators and higher inflammatory factors in group H.

The phyla of Tenericutes cannot produce the anti-oxidant, indole propionic acid and the increase in its abundance reduces antioxidant ability and evokes inflammation in the chorioamniotic membrane (Doyle et al., 2017; Menni et al., 2019). Patescibacteria is a phylum associated with a number of viruses (Flint et al., 2012), and a decrease in its abundance enhances the antioxidant ability in elderly people (Zhang et al., 2021). The low antioxidant indicators and nutrient digestibility, as well as, the great levels of inflammatory factors in group L were strongly associated with the higher abundances of Tenericutes and Paterscibacteria in this study. Further analysis of the differences at the family and genus levels found that the families of norank_f_norank_o_Mollicutes_RF39 and Saccharimonadaceae and genera of *norank_f_norank_o_Mollicutes_RF39* and *Candidatus_Saccharimonas* were significantly enriched in group L compared to group H and M, and were the main reason for the increased abundances of Tenericutes and Paterscibacteria. Moreover, the genus of *norank_f_norank_o_Mollicutes_RF39* was positively correlated with the concentrations of IL-2 and IL-6, and the genus of *Candidatus_Saccharimonas* was negatively correlated with CAT, T-SOD, and GSH-Px activity.

Compared to group H and M, the phylum of Bacteroidetes in group L was numerically increased, and the family of Rikenellaceae and the genus of *Rikenellaceae_RC9_gut_grou*p were significantly enriched in group L. Previous studies have found that *Rikenellaceae* was highly correlated with the lean body mass (BMI < 25) in humans (Flint et al., 2012). Similar results were obtained in this study with the genus *Rikenellaceae_RC9_gut_group* being inversely correlated with CP digestibility, ADG, and serum CAT activity. The aforementioned finding suggests that the lower antioxidant ability and nutrient digestibility, as well as the greater inflammatory responses of donkey jennets on the L diet may be the result of increases in *norank_f_norank_o_Mollicutes_RF39*, *Candidatus_Saccharimonas*, and *Rikenellaceae_RC9_gut_group*.

Fibrobacteres is the main ligninolytic phylum and is closely correlated with antioxidant abilities in animals. Studies in piglets have shown a positive correlation between Fibrobacteres with both T-AOC and CAT activity in the jejunum (Xu, 2021; Xu et al., 2021). In this study the Fibrobacteres phylum, the family of Fibrobacteraceae and genus of *Fibrobacter,* were increased in the pregnant jennets on the M diet compared to group H and L. Furthermore, the abundance of *Fibrobacter* was positively correlated with serum CAT and T-SOD activity and inversely correlated with IL-2 concentration.

Additionally, phylum of Firmicutes in the group M also increased numerically compared to group H and L, and the families of Achiminococcaceae and Clostridiaceae_1 and genera of *Phascolarctobacterium* and *Clostridium_sensustricto_1* were enriched. *Phascolarctobacterium* produces organic acids that can inhibit *Escherichia coli* and regulate the gastrointestinal health (Xu et al., 2021). The decrease in the abundance of *Clostridium_sensustricto_1* is often a marker of intense inflammation, and a significant decrease in the abundance of *Clostridium_sensustric-to_1* has been found in patients with immunoglobulin A nephropathy and membranous nephropathy (Dong et al., 2020). In this study, the abundance of *Phascolarctobacterium* was positively correlated with CAT activity in serum, and *Clostridium_sensustricto_1* was positively correlated with CP, ADF and NDF digestibilities, as well as serum CAT and GSH-Px concentrations, but negatively correlated with the MDA concentration. The above results show that the great antioxidant status of pregnant jennets on the M diet was closely related to the abundances of these bacteria, with Fibrobacter playing a key role.

The results of this experiment demonstrates that dietary energy level can affect pregnant jennets’ health by altering the gut microbial community. Previous studies have found that energy intake indirectly changes the amount of NO produced by altering amino acid metabolism. Nitrites and nitrates oxidized by NO affect the composition and function of the microbiota (Alemany, 2013). It is thus proposed that dietary energy level may further affect the composition of intestinal microflora by influencing amino acid metabolism, and these specific mechanisms need to be further exploration.

In addition, this experiment found that as the parturition approached, mobilization of body fat in the body increased, aggravating the physiological function of the liver with a significant increases in NEFA and BHBA concentrations, especially within the 7 days before parturition. This exacerbated the oxidative stress and inflammatory responses of the pregnant jennets, as indicated by a decrease in antioxidant enzyme activity and an increase in inflammatory factors. Meanwhile, the relative abundance of *Lachnospiraceae_NK4B4* increased and *norank_f_Bacteroidales_UCG-001* decreased, apparently being associated with changes in the energy metabolism of the host. Therefore, it is critical to ensure that an appropriate level of dietary energy is available for pregnant donkeys during late gestation, and that the feeding and management within 7 days before the parturition deserves special attention.

## 5 Conclusion

In conclusion, dietary energy level of 10.49 MJ/kg for pregnant donkeys during late gestation increased the antioxidant capacity, reduces inflammatory cytokines, and promoted nutrients digestion, and fetal growth. These changes were associated with the alterations in the rectal microbiota composition. Superfluous or insufficient dietary energy supply during late gestation led to an increase of oxidative stress and inflammation in pre-partum donkeys.

## Data availability statement

The datasets presented in this study can be found in online repositories. The names of the repository/repositories and accession number(s) can be found in the article/Supplementary material.

## Ethics statement

The animal studies were approved by all animal procedures were performed under the national standard Guidelines for Ethical Review of Animal Welfare (GB/T 35892-2018). The studies were conducted in accordance with the local legislation and institutional requirements. Written informed consent was obtained from the owners for the participation of their animals in this study.

## Author contributions

YG: Conceptualization, Funding acquisition, Investigation, Project administration, Resources, Validation, Visualization, Writing – original draft. GY: Data curation, Formal analysis, Investigation, Methodology, Writing – original draft. FH: Data curation, Formal analysis, Investigation, Writing – original draft. XG: Writing – review & editing. BS: Supervision, Writing – review & editing. YZ: Writing – review & editing, SY: Conceptualization, Project administration, Resources, Supervision, Writing – review & editing.
