# Supplementary material for: Effects of dietary energy level on antioxidant capability, immune function and rectal microbiota in late gestation donkeys
Source: Front Microbiol. 2024 Feb 13;15:1308171. doi: 10.3389/fmicb.2024.1308171 (PMC10896733; doi:10.3389/fmicb.2024.1308171)
Supplement: Supplementary file 1 [file Table_1.DOCX]

**Table S1** Effects of dietary energy level on nutrient digestibility (%) of donkey jennets during late gestation

|  | H | M | L | SEM | *P*-value |
| --- | --- | --- | --- | --- | --- |
| **d-35** |  |  |  |  |  |
| DM | 55.67^A^ | 57.12^A^ | 47.95^B^ | 0.891 | <0.001 |
| CP | 77.06^B^ | 79.49^A^ | 75.20^C^ | 0.308 | <0.001 |
| EE | 66.99^B^ | 69.72^A^ | 55.71^C^ | 0.551 | <0.001 |
| ADF | 36.63^A^ | 39.20^A^ | 27.37^B^ | 1.043 | <0.001 |
| NDF | 37.57 | 40.96 | 32.55 | 2.176 | 0.064 |
| **d-7** |  |  |  |  |  |
| DM | 57.52 | 52.90 | 53.91 | 1.557 | 0.143 |
| CP | 76.92 | 79.62 | 76.52 | 0.998 | 0.109 |
| EE | 60.22 | 57.30 | 58.91 | 3.606 | 0.851 |
| ADF | 38.18 | 45.21 | 41.60 | 3.007 | 0.303 |
| NDF | 41.82 | 47.76 | 42.05 | 2.512 | 0.220 |

^CP: crude protein; DM: dry matter; EE: ether extract; ADF: acid detergent fiber; NDF: neutral detergent fiber.^

^H, M, and L refer to high digestible energy (10.92 MJ/kg), medium energy (10.49 MJ/kg), and low energy (9.94 MJ/kg) of the diets.^

^d-35, d-7: days before foaling.^

^A-D Means with different superscripts within a row differ significantly (P ≤ 0.05)^
